# A Novel Inhibitor of Translation Initiation Factor eIF5B in *Saccharomyces cerevisiae*

**DOI:** 10.4014/jmb.2404.04015

**Published:** 2024-06-05

**Authors:** Ah-Ra Goh, Yi-Na Kim, Jae Hyeun Oh, Sang Ki Choi

**Affiliations:** Department of Biomedical Sciences, Sunchon National University, Sunchon 57922, Republic of Korea

**Keywords:** Translation inhibitor, translation initiation factor, eIF5B, antifungal, target

## Abstract

The eukaryotic translation initiation factor eIF5B is a bacterial IF2 ortholog that plays an important role in ribosome joining and stabilization of the initiator tRNA on the AUG start codon during the initiation of translation. We identified the fluorophenyl oxazole derivative 2,2-dibromo-1-(2-(4-fluorophenyl)benzo[d]oxazol-5-yl)ethanone quinolinol as an inhibitor of fungal protein synthesis using an in vitro translation assay in a fungal system. Mutants resistant to this compound were isolated in *Saccharomyces cerevisiae* and were demonstrated to contain amino acid substitutions in eIF5B that conferred the resistance. These results suggest that eIF5B is a target of potential antifungal compound and that mutation of eIF5B can confer resistance. Subsequent identification of 16 other mutants revealed that primary mutations clustered mainly on domain 2 of eIF5B and secondarily mainly on domain 4. Domain 2 has been implicated in the interaction with the small ribosomal subunit during initiation of translation. The tested translation inhibitor could act by weakening the functional contact between eIF5B and the ribosome complex. This data provides the basis for the development of a new family of antifungals.

## Introduction

Infections caused by opportunistic fungal pathogens such as *Candida albicans* in immunocompromised patients has increased substantially. Many pathogenic fungi tend to be resistant to antifungals, despite the ongoing development of antifungal drugs [1]. The development of novel drugs based on new fungal targets is crucial. To this aim, the translation system is attractive, being composed of many components including mRNA, tRNAs, ribosomes, and many protein factors that could be useful as antifungal targets.

The eukaryotic initiation factor IF2 ortholog eIF5B is universally conserved [2]. eIF5B is recruited to the small 40S ribosomal subunit and promotes binding to the large 60S subunit [3]. Formation of the 80S ribosome causes eIF5B to hydrolyze GTP since eIF5B is a ribosome-dependent GTPase, and causes the release of eIF1A, which subsequently binds to eIF5B from the ribosomal A site [3, 4]. eIF5B contains conserved central GTP binding and C-terminal regions [5]. The eIF5B N-terminal region is non-essential, and N-terminally truncated ΔeIF5B_397–1002_ is fully active [5]. The X-ray determined structure of the aIF5B of the archaeal organism *Methanobacterium thermoautotrophicum* resembles a chalice, in which the G domain (domain 1), domain 2 and domain 3 form a globular cup that is connected to domain 4 to form a base via a stem-like helix H12 [6]. Binding of eIF5B to GTP produces modest conformational changes that trigger a swing of helix H12 and larger movement of domain 4 [2, 7]. Results from directed hydroxyl radical cleavage studies have suggested that eIF5B is located in the intersubunit cleft of the 80S ribosome with domain 1 positioned near the GTPase activating center of the 60S subunit and with domain 2 interacting with helix h5 of 18S rRNA and ribosomal protein rpS23 of the 40S subunit [8, 9].

We previously developed sensitive in vitro translation system in *Saccharomyces cerevisiae* [10]. This in vitro translation assay could be used to monitor the activity of translation in the presence of an unknown compound. The advantage of this biochemical inhibition assay is that multiple translation components playing a role in protein synthesis can be targeted by a putative inhibitor, greatly increasing the chance of identifying inhibitor(s) that could be developed as a novel anti-fungal.

In the present study, we analyzed the in vitro antifungal activities of three compounds – fluorophenyl oxazole derivatives (2-(4-fluorophenyl)-7,8-dihydronaphtho[2,3-d]oxazol-5(6H)-one, 3-[2-(4fluoro-phenyl)-6-methyl-benzooxazol -5-yl]-3-oxo-propionic acid methyl ester and 2,2-dibromo-1-(2-(4-fluorophenyl)benzo[d]oxazol-5-yl)ethanone) – against pathogenic fungi. To develop these translational inhibitors as antifungal agents, we attempted to find a molecular target of the compounds using resistant mutants as well as identification of the resistant alleles. It was demonstrated that amino acid substitutions in eIF5B conferred the resistance, and that the majority of the resistant mutants involved amino acid changes in domain 2 and domain4 of eIF5B.

## Materials and Methods

### Chemicals and Media

Approximately 2,300 core chemicals and 80 chemical derivatives of 2-(4-fluorophenyl)-7,8-dihydronaphtho[2,3-d]oxazol-5(6H)-one (290HR0015) were obtained from Korea Chemical Bank. 2,2-Dibromo-1-(2-(4-fluorophenyl)benzo[d]oxazol-5-yl)ethanone (1282NJ0069) was further synthesized and kindly provided by Dr. K.Y. Lee, Korea Research Institute of Chemical Technology. *Candida albicans*, *Saccharomyces cerevisiae*, *Candida glabrata*, *Candida lusitaniae*, and *Cryptococcus neoformans* were grown on RPMI1640 basal medium supplemented with 2% glucose buffered to pH 7.0 with 0.165 M morpholinepropanesulfonic acid (Sigma-Aldrich, USA) to measure minimum inhibitory concentration (MIC) for the chemical. Chemical resistant mutants that grew on SC media were used to determine the MIC for the chemical.

### Strains, Growth Conditions, and Plasmids

Wild type haploid *S. cerevisiae* strain H1515(*MATa trp1-Δ63ura3-52leu2-3leu2-112*) was used in the selection of 1282NJ0069 resistant mutants. The yeast-*Escherichia coli* micron based shuttle vector pRS426 was used to construct the 1282NJ0069 resistant mutant libraries. For yeasts, a 96-well plate was used to prepare microdilution panels containing chemicals in 0.1 ml of medium, with concentrations ranging from 0.01–50 μg/ml. The starting inocula were adjusted by the spectrophotometric method to a final OD_600_ of 0.01. Then, each well was inoculated with 10 μl of the adjusted yeast suspension. The inoculated plates were incubated at 30° C without agitation for 12–36 h. Following incubation and after agitation with a microtiter plate shaker for 5 min, the plates were read spectrophotometrically with an automatic plate reader set at 600 nm. Yeast transformations were done by the electroporation as described previously [11]. *E. coli* DH5α [*endA1 hsdR1 supE44 thi-1 recA1 gyrA9 relA1* Δ*lacU169*(j80*lacZΔM15*)] was used for transformation and preparation of plasmid DNA. All DNA manipulations were carried out by standard procedures [12].

### Isolation of Resistant Mutants

*S. cerevisiae* strain H1515 was grown overnight in YPD broth at 30°C to about 1.0 × 10^8^ cells/ml. Cells were concentrated by centrifugation at 6000 ×*g* for 5 min and resuspesion in 50 mM potassium phosphate buffer, pH 7.0. A 300 μl aliquot of ethyl methanesulfonate (EMS) was added to 10 ml of approximately 5 × 10^7^ cells/ml of *S. cerevisiae* strain H1515 in 50 mM potassium phosphate buffer, pH 7.0, and then incubated for 30 min at 30°C with shaking [13]. The EMS was inactivated by the addition of 10% (w/v) sodium thiosulfate (Sigma-Aldrich). The cells were washed twice with sterile water and plated out on SC agar plates containing 5 μg/ml of 1282NJ0069. Resistant mutants appeared after 2 days of incubation at 30°C. The frequency of resistance was determined by dividing the number of the mutants by the total number of cells plated. The MIC values of the resulting mutants were determined in RPMI medium using the microbroth dilution method [14].

### Construction of Plasmid Genomic DNA Library and Identification of the Compound A Resistance Gene

Genomic DNA isolated from resistant mutant PSY125 was sheared with a HydroShear^®^ device (GeneMachines, USA) and fractionated to collect sheared DNA above 4 kb using Chromaspin 10 columns (Clontech, USA). DNA was concentrated by ethanol precipitation. Single stranded ends of the DNA were filled, phosphorylated and ligated with Blunt kination ligation kit (Takara Bio, Japan). The DNA was ligated into pRS315, digested with *Sma*I, purified by successive extractions with phenol/chloroform/isoamyl alcohol (25:24:1) and chloroform/isoamyl alcohol (24:1) extraction and ethanol precipitated. The DNA was used to transform *E. coli* DH5α electrocompetent cells via electroporation. Plasmid DNA was extracted from pooled *E. coli* samples and was used to transform *S. cerevisiae* BY4741 using a Gene Pulser electroporator (BioRad, USA). Resistance to 16.5 μg/ml of 1282NJ0069 and uracil prototrophy was carried out on uracil-deficient synthethic defined medium. DNA was extracted from the 1282NJ0069 resistant colonies using a DNeasy kit (Qiagen, USA) and used to transform *E. coli*. Plasmid DNA was extracted from the ampicillin resistant *E. coli* colonies and transformed again into the yeast strain H1515 to demonstrate plasmid-dependent resistance to 1282NJ0069. If the plasmid conferred 1282NJ0069 resistance to H1515, the plasmid was digested with *Sal*I/*Sac*I, the size of the inserted DNA was checked by gel electrophoresis and sequenced (Genotech, Republic of Korea) using M13 universal primers. Additional sequencing of eIF5B DNA was performed using primers 5'- AGA GCA CAT GTG CAT GAA GT-3' and 5'- GTG AAC TAC GTT TGA AAT-3' to identify base substitutions that conferred resistance on *S. cerevisiae* strain H1515 upon transformation.

### Identification and Cloning of eIF5B Mutations

Genomic DNA of 20 1282NJ0069 resistant mutants was isolated and sequenced using the forward primer 5’-AGA GCA CAT GTG CAT GAA GT-3’ and reverse primer 5’- GAC AAG TCA GCG TAT GCC-3’. The base substituted eIF5B was amplified using primers 5’- CTC CAT CCA GCG CTT CTC CA-3’ and 5’- CCC CCG AGC TCG AGA CTA ATA CAC AAA GGT TCA C-3’, which contained SacI and XhoI sites. The PCR product was digested with *Eco47*III/SacI and ligated to *Eco47*III/SacI digested pC691 containing the eIF5B gene in pRS316. The cloned plasmid was distinguished with pC691 by identifying whether the plasmid retained the XhoI site. The 1282NJ0069 resistant mutant was PCR amplified using the aforementioned primers. The mutant allele in plasmid was transformed to either H1515 or The *eIF5BΔ* strains J130 (*MATa ura3-52 leu2-3 leu2-112 fun12::hisG*) and tested growth on media with or without inhibitor [15].

### MIC_50_ Determination

**Fig. 1:**
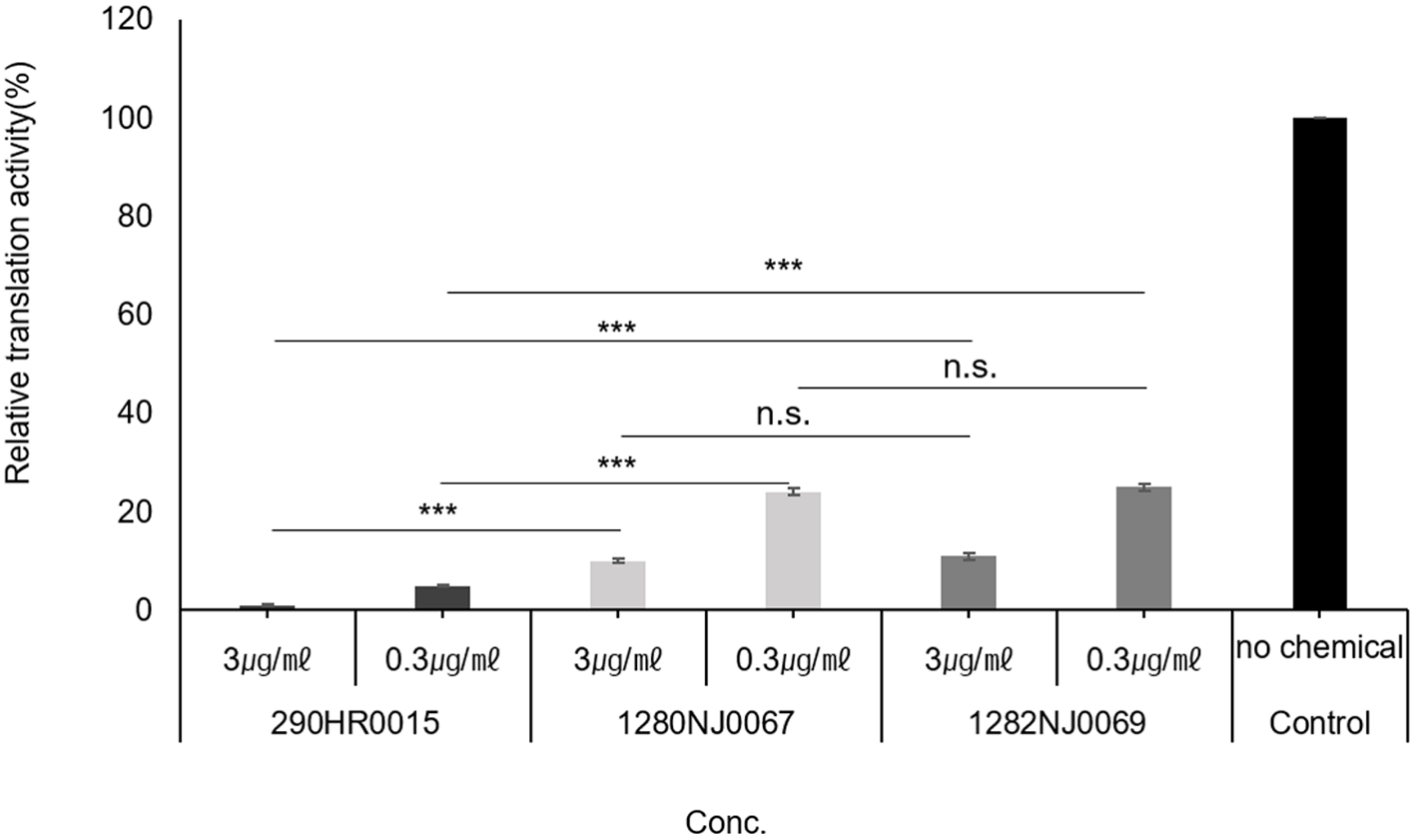
In vitro translation activities in the presence of inhibitors. Crude yeast S30 extracts were prepared from strain H1515. In vitro translation products from mRNA encoding the LUC protein were analyzed by luminescence. Data are expressed as the percent of LUC protein produced in extracts treated with 0.3–3.0 μg/ml of chemical. The results obtained from each experiment were expressed as mean and standard deviation SD) in triplicate. *** *p* < 0.001, ** *p* < 0.01, * *p* < 0.05, and n.s. stands for not significant.

The MICs of the examined antifungal compounds were determined by a whole-cell assay in a 96-well plate format. Fungi with an initial cell optical density at 600 nm (OD_600_) of 0.01 in RPMI-MOPS medium were inoculated with the medium containing the chemicals serially diluted 2-fold from 0.01–100 μg/ml, followed by incubation at 30°C. SC medium was used for growing 1282NJ0069 resistant mutants. Growth inhibition was measured by determining the OD_600_ at 24–36 h. The lowest concentration that caused 50% growth inhibition represented the MIC_50_ of the compound. Stock solutions of 1282NJ0069 and other derivatives were dissolved in dimethyl sulfoxide (DMSO) at 5 mg/ml. The chemical structures of representative 1282NJ0069 used in the main experiments are shown in Fig. 1. Commercial antifungal agents itracoazole and ketocoazole were obtained from Sigma-Aldrich and were dissolved at 2.5 mg/ml in DMSO.

### In vitro Translation Assays

Translation assays were performed as described previously [10]. Cytoplasmic S30 extracts were prepared essentially as described previously [16] and stored in liquid N_2_. The extracts were thawed on ice and mixed with 2× translation buffer (40 mM HEPES pH 7.4, 260 mM potassium acetate, 4 mM magnesium acetate, 1.5 mM ATP, 0.2 mM GTP, 3 mM dithiothreitol, 50 mg/ml creatine phosphate, 0.3 mg/ml creatine phosphate kinase, 0.08 mM amino acids). Capped and polyadenylated luciferase mRNA was prepared with the T_7_ luciferase (T_7_LUC) vector and T_7_ transcription kit. mRNA was purified with the RNeasy total RNA kit.

The chemical samples were diluted 100 times with distilled water and added to 3 μl of assay reaction to make a final 15 μl volume including translation extract and luciferase mRNA, and then incubated for 30 min at 25°C. Reactions were quenched by quick freezing in liquid N_2_. After thawing on ice, luminescence was measured by adding 10 μl of the translation mix to 50 μl of LUC assay reagent (Promega, USA) and measuring the emission for 15 sec on a LMax Microplate Luminometer (Molecular Devices, USA). All presented data are representative of at least three independent experiments.

## Results

### Identification of the Fluorophenyl Oxazole Derivatives 2,2-Dibromo-1-(2-(4-fluorophenyl)Benzo[d]Oxazol-5-yl)Ethanone Quinolinol (1282NJ0069) as an Antifungal Compound

We established an in vitro translation system of a luciferase mRNA reporter that enabled us to identify translation inhibitors. To maximize the sensitivity of the in vitro translation reaction for detection of protein synthesis, a final mRNA concentration was determined to be 50 ng/μl and reaction was incubated for 30 min, which was a first order reaction at 25°C. To validate that this reaction was appropriate for screening translation inhibitors, we chose a set of known inhibitors representing different activities and proved that the translation reaction was inhibited with different efficiencies as determined by the concentration of the compound that caused 50% inhibition with respect to the compound-free control. Hygromycin, puromycin, and cycloheximide yielded IC_50_s of 0.01, 1.0, and 1.0 μg/ml, respectively [10].

We screened 3,200 core chemicals and approximately 80 derivatives provided by the Korea Chemical Bank and identified 2-(4-fluorophenyl)-7,8-dihydronaphtho[2,3-d]oxazol-5(6H)-one (290HR0015) as a core compound, and 3-[2-(4-fluoro-phenyl)-6-methyl-benzooxazol-5-yl]-3- oxo-propionic acid methyl ester (1280NJ0067), 2,2-dibromo-1-(2-(4-fluorophenyl)benzo[d]oxazol-5-yl)ethanone (1282NJ0069) as its derivative, as inhibitors of in vitro protein synthesis and significant growth reduction of pathogenic fungi (Table 1). An in vitro translation assay was performed in the presence of increasing concentrations of the chemicals. These compounds inhibited translation in vitro proportionally as concentration increased from 0.3–3 μg/ml (Fig. 1). To define the spectrum of action of these new antifungal agents, the in vitro activities of these chemicals against a wide range of pathogenic yeasts were evaluated. MIC of 1282NJ0069 for *Candida albicans*, *C. glabrata*, and *C. lucitaniae* was 0.25, 0.5, and 0.5 μg/ml, respectively, which represented a 4-fold higher IC_50_ than 290HR0015; the MIC_50_ of the compound for Candida was lowered significantly. These MICs were almost equivalent with those of itraconazole and ketoconazole as controls. The data indicate that the chemical is an effective inhibitor of protein synthesis in fungi.

### Isolation of 1282NJ0069 Resistant Mutants

It has been successfully demonstrated that mutants resistant to antifungal compounds display activity of the target [17]. With this knowledge, we adopt this strategy to identify the molecular target of 1282NJ0069. *S. cerevisiae* H1515 cells were mutagenized with EMS and plated on medium containing 5 μg/ml of the test chemical. Mutants isolated were further checked for their growth on SC media containing 10 μg/ml of the chemical. Twenty mutants were discovered. To verify that the mutation is dominant to the wild-type allele, the resistant haploid mutant strain was crossed to the wild-type haploid strain. The resulting diploid also displayed a 4-fold increase in MIC_50_ compared to the wild-type parental diploid strain, indicating that the resistance conferred by the mutant gene is dominant to its wild-type allele. *S. cerevisiae* was 15 times more sensitive than *C. albicans* to 1282NJ0069, probably due to different media used (SC for Saccharomyces and RPMI for Candida). The mutants selected were at least 6 times more resistant than the wild type to the compound (Table 2). Growth of mutants, especially PSY75, PSY125, and PSY76, was better than that of wild type.

### Mutation in eIF5B Confers Resistance to 1282NJ0069

To identify the mutated gene, a genomic DNA library was constructed from the resistant mutant PSY125, which was identified to be dominant to the wild-type allele. The library was transformed into *S. cerevisiae* strain H1515 and screened for colonies resistant to 1282NJ0069.

**Fig. 2:**
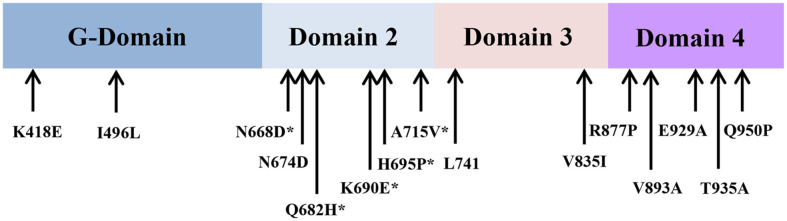
The amino acid residues changed in eIF5B identified in 1282NJ0069 resistant mutants. Mapping of the PSY125 mutations on the primary structure of eIF5B. Position and nature of the amino acid change in the sequenced mutant alleles of eIF5B, and the amino acid residue found in the resistant mutant is shown as arrow below the line. Star indicates single residues changed in mutations representing the primary position showing resistance to the chemical.

Screening of approximately 23,000 *S. cerevisiae* colonies transformed with the library DNA yielded six 1282NJ0069 resistant isolates. Plasmids recovered from the six resistant transformants were transformed into the *S. cerevisiae* strain H1515. The resulting resistant transformants indicated that resistance was due to the plasmid. Plasmids conferring 1282NJ0069 resistance were recovered, and analyzed to identify the common regions and the full sequence capable of conferring resistance. There were 2.5, 2.7, 2.9, 3.0, two 4.0-kb SalI/SacI fragments, which was then fully sequenced. The sequences of the two 4-kb DNA fragments obtained matched that of *eIF5B*, a gene encoding a 1002-amino acid protein. A single base change of C to T at base 2144 of eIF5B in the plasmid caused an amino acid substitution of A to V at residue 715 in domain 2, which was conserved as neutral amino acid among eIF5B from different eukaryotes (Fig. 2). Although the function of the region is not unclear, our data show that the A715V mutation leads to resistance of *S. cerevisiae* cells to 1282NJ0069. Although the other four DNA fragments comprised full or partial sequences of rRNAs, no sequences were changed. It seemed that rRNA overexpressed in the 2-micron plasmid conferred resistance to the strain.

To discover if the mutational hot spot of eIF5B conferred resistance to the chemical, we amplified and sequenced the functional region, with the exception of the N- terminal region in *eIF5B* from genomic DNA prepared from the 20 other resistant mutants. These mutants displayed 6–34 times greater MIC_50_ compared to the control *S. cerevisiae* strain H1515. Single base changes that resulted in amino acid substitutions in each mutant were identified in four other mutants (PSY75, GFL6, GFL16, GFL107 and GFL124). Other mutants were composed of several base changes that resulted in several substitutions of 2–5 amino acids of eIF5B (Table 2). The amino acid changes conferring resistance to 1282NJ0069 clustered into three regions of the eIF5B protein (Fig. 2).

Amino acid sequence alignment of eIF5B with its prokaryotic counterpart IF2 demonstrated that the eIF5B single base substitutions were mainly located in regions with homology to domains 2 in IF2. These domains of eIF5B are thought to interact with ribosome during ribosome joining and initiator Met-tRNA stabilization on AUG. We propose that translation initiation would not normally proceed because of the binding of 1282NJ0069 at the interface between ribosome and eIF5B, while mutations in eIF5B conferring resistance to 1282NJ0069 are located in the domain that contacts the ribosome (Fig. 2). Since a 2-micron plasmid was used for cloning, it was possible that the resistance was due to overexpression of wild-type eIF5B. To eliminate this possibility, the mutant *eIF5B* gene was also cloned into a centromeric vector pRS316, whereas the wild-type *eIF5B* genes were cloned into 2-micron plasmid pRS426, and then this plasmid was transformed into strain H1515. MIC testing of the transformants showed that only the strain expressing the mutant *eIF5B* was resistant to 1282NJ0069. This demonstrates that a mutation in *eIF5B* confers resistance. Although the function of region is not unclear, our data show that the A715V mutation leads to resistance of *S. cerevisiae* cells to 1282NJ0069.

### Most Primary Resistance Mutations Cluster on a Domain 2 of eIF5B

*S. cerevisiae* eIF5B displayed 53% homology to bacterial IF2, and it presumably folded into the same structural and functional domains [1, 6]. Localization of resistance mutations within the eIF5B protein could give clues to the mechanism of resistance and, hence, to the mode of action of 1282NJ0069. The nature of the amino acid changes is shown in Table 2. The 15 altered positions on *eIF5B* include six primary mutated residues clustered on domain 2 and five mapping to domain 4.

Fig. 2 depicts the six clustered single mutations in domain 2 in the context of the sequences of other eukaryotic eIF5B proteins. The nature of the amino acid substitutions did not give obvious clues to the mechanism of resistance, but the sequence alignment showed there location in domain 2. It is therefore surprising how an amino acid change of eIF5B could confer tolerance to the harmful chemical and yet retain functionality of the protein. The N668D and K690E mutations introduced an acidic residue at a position where none is found naturally. Another unusual change was introduced by the H695P mutation. Yet, the resistant mutants grew at a rate that was not significantly different from that of the wild type in the absence of the drug (Table 2).

Interestingly, the G domain mutant strains PSY75 and PSY76, and one domain 2 mutant strain, PSY125, showed faster growth compared to the normal strain. Each mutant strain had various MIC_50_ values, but did not show a direct relationship with growth.

### Introduction of PSY76 Mutant Allele to Wild Type Strain Confers Resistance to the Inhibitor

**Fig. 3:**
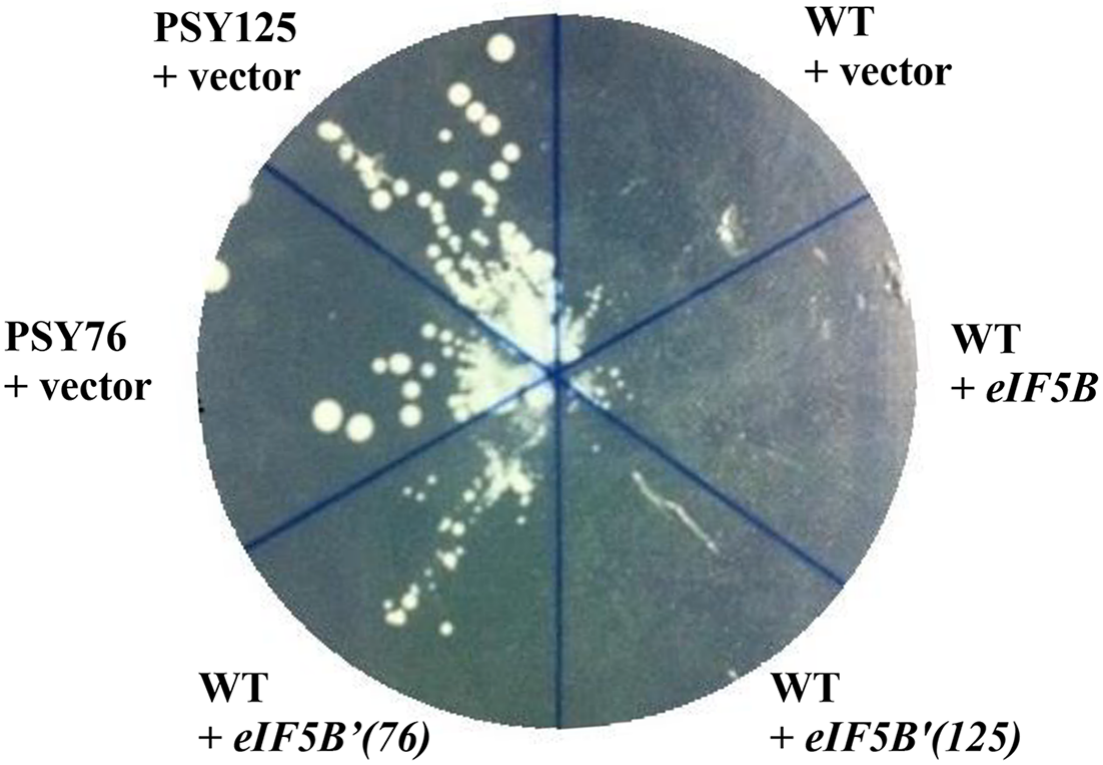
Overexpression of PSY76 mutant allele to wild type strain confers resistance to the inhibitor. Overexpression of PSY76 mutant alleles in wild type strain restores growth on media containing inhibitor. PSY76 mutant and PSY125 mutant strains was transformed with the high–copy number plasmid or with vector carrying mutant allele each. Transformants were streaked on minimal SD medium supplemented only with the required nutrients and inhibitor, and then incubated 5 days at 30°C.

The characteristics of the PSY76 and PSY125 mutant strains, which showed relatively fast growth, were investigated. Fig. 3 shows the growth of the normal strain and the mutant strain in which the PSY76 mutant allele was inserted into a high-expression vector in a normal strain on a medium supplemented with the compound. The strain expressing mutant *eIF5B*'(76) allele in a normal strain showed resistance to the compound, though that was less resistance than the compound-resistant mutant strain. However, the strain expressing *eIF5B*'(125) allele did not show resistance.

**Fig. 4:**
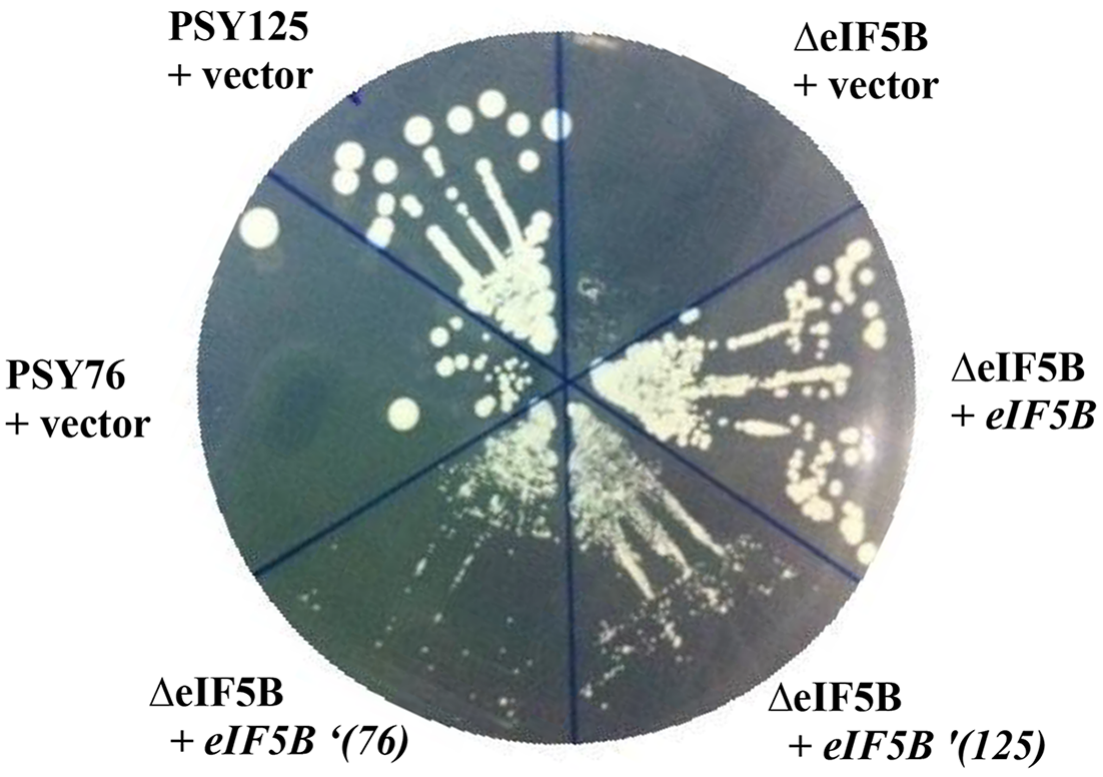
Overexpression of PSY76 mutant allele to eIF5BΔ strain partially restores slow-growth phenotype. PSY76 mutant and PSY125 mutant strains was transformed with the high–copy number plasmid or with vector carrying mutant allele to *eIF5BΔ* strain. Transformants were streaked on minimal SD medium supplemented only with the required nutrients, and then incubated 5 days at 30°C.

Fig. 4 shows changes in growth after overexpression of mutant *eIF5B*' (76 or 125) allele in a strain with deleted eIF5B in a medium without added compounds. It can be observed that overexpression of mutant *eIF5B*' (76 or 125) allele partially restores the function of the normal gene, eIF5B. It seems that overexpression of the mutant alleles would be toxic for cell growth since the mutant stains grow as wild type.

These results show that the I496L and V835I double mutant strains play a partial role in resistance to compounds. However, the A715V mutant strain does not show resistance to the compound, but shows some of the functions of this gene for growth in the absence of the compound, so it is assumed that overexpression of the mutant gene in wild type does not show toxicity to the normal gene. The current results also show that the eIF5B gene was targeted by a compound isolated in this laboratory and caused a mutation, but judging from the results in Figs. 3 and 4, this resistance is likely not limited to protein synthesis factors but also to members such as small ribosomes that play a role together.

## Discussion

The ribosomal protein synthesizing machinery is thought to be highly conserved among eukaryotic organisms [18]. The translation machinery of eukaryotes and prokaryotes evolved differently from each other. Thus, protein synthesis inhibitors could be good antimicrobial compounds, but are unlikely to be selective inhibitors of eukaryotic microbes because eukaryotic genes are highly homologous among organisms. Whereas three translation initiation factors have been identified in prokaryotes, translation initiation in eukaryotes requires at least 12 independent factors [19]. IF1/eIF1A and IF2/eIF5B are conserved in all three kingdoms. Surprisingly, we found that *S. cerevisiae* eIF5B was a target of potential antifungal 1282NJ0069, which was identified as an inhibitor of translation initiation in vitro. Eighteen of 20 alleles sequenced in the resistant mutants changed and clustered primarily at domain 2 and secondarily at domain 4.

Domain 2 is a β-barrel consisting of eleven antiparallel β-strands, arranged in the following order: S9–S10–S11–S12–S13–S14–S15–S16–S17–S18–S19 [6]. Most of the primary mutations resistant to the chemical were found to be located in β strands in domain 2 except one residue, L741S in H8, of which N668D, Q682H, K690H, H695P, and A715V were between S13 and S19. Three β strands (S10, S11, and S19) are responsible for stabilizing the relative orientations of domains 1 and 2 through interaction with the switch region of G domain [6]. However, most of the residues responsible for the resistance to the chemical were not in those positions.

In vitro chemical probing studies using tethered nucleic acid cleavage agents and footprinting techniques to introduce cleavages in the rRNA supported the findings of the cryo-electron microscopy studies that revealed that the IF2 and eIF5B G domains contact the large ribosomal subunit and that the eIF5B domain 2 contacts the small subunit [6, 20, 21].

All unmodified and conjugated domain 2 mutants of human eIF5B displayed wild-type activity in the methionyl-puromycin synthesis assay; G domain mutants were fully active. All unmodified domain 3 mutants had wild-type activity [9].

Recent experiments with mutant alleles of domain 2 of human and yeast eIF2B indicate that domain 2 interacts with the 40S subunit [9]. Therefore, primary mutants in the domain 2 (N668D, Q682H, K690E, H695P, A715V, and L741S) would be in contact with the ribosome. This report supports the view that the domain 2 region is mutated, which confers resistance against the chemical through contact with small ribosome.

There were two interesting characteristics of G-domain mutant. GTP hydrolysis, but not subunit-joining activity, is impaired in vitro, but cannot release eIF5B from ribosome and exhibits a severe slow-growth phenotype. Secondly, GTPase activity is impaired, which lowers eIF5B ribosome binding affinity, enabling the factor to dissociate from the 80S ribosome and almost restore wild-type growth [8].

Secondary mutations were clustered in the C-terminal end between S25 and S31 in domain 4 (Fig. 2). Domain 4 interacts with tRNA and /or eIF1A [22]. It is possible that mutation in domain 4 assists the effect of primary mutation in domain 2 to the chemical. GFL76 and GFL101 had several mutations (V893A, E929A, T935A, R877P, and Q950P) in domain 4 in addition to Q682H primary mutation in domain 2, and lowered MIC_50_, which indicates that the mutants became more sensitive to the chemical. Interestingly, growth of the mutants was comparable to that of the wild-type, although there were mutations in both domain 2 and domain 4.

Given the number of isolates analyzed so far, the mutant screen cannot be considered saturated. Thus, the pathway inhibited by 1282NJ0069 could still contain additional components, and these presumably could be involved in the eIF5B function and its interacting ribosomal component. Thus, 1282NJ0067 inhibitors can also be useful tools in exploring fine function of eIF5B as well as in identification of the ribosomal components involved.

In summary, using in vitro translation assay, we screened a large collection of compounds, resulting in the identification of several new translation inhibitors. A new chemical class of inhibitors was also identified as having in vivo activity. it was found that eIF5B is a target of the antifungal compound and its mutation can confer resistance. In future, structure-activity relationship studies with this series of compound could lead to the identification of compounds with potent translation inhibition activity.

## Figures and Tables

**Table 1:** Structures and IC_50_s of 290HR0015 and two derivatives identified in the screen of translation inhibitor.

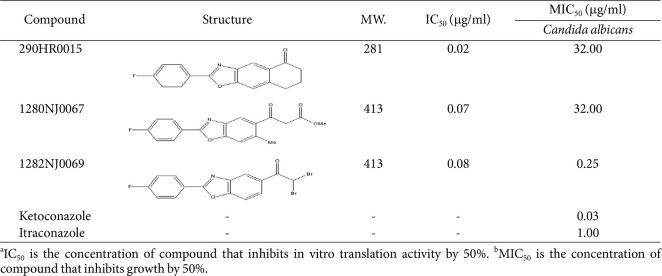

**Table 2:** Summary of eIF5B mutations.

Mutants	Nucleotide	Amino acid residue	MIC_50_(μg/ml)	Growth
PSY75	1486(A→C)	496(I→L)	0.35	+++
PSY125	2144(C→T)	715(A→V)	0.43	+++
GFL 6	2046(A→C)	682(Q→H)	0.55	++
GFL16	2068(A→G)	690(K→E)	0.29	++
GFL107	2002(A→G)	668(N→D)	0.31	++
GFL124	2084(A→C)	695(H→P)	0.10	++
PSY76	1486(A→C)	496(I→L)	0.23	+++
	2503(G→A)	835(V→I)		
GFL 4	2020(A→G)	674(N→D)	0.20	++±
	2068(A→G)	690(K→E)		
GFL17	1252(A→G)	418(K→E)	0.54	++
	2222(T→C)	741(L→S)		
GFL52	2020(A→G)	674(N→D)	0.14	++
	2046(A→C)	682(Q→H)		
GFL128	2222(T→C)	741(L→S)	0.37	++±
	2678(T→C)	893(V→A)		
	2849(A→C)	950(Q→P)		
GFL76	2046(A→C)	682(Q→H)	0.21	++
	2678(T→C)	893(V→A)		
	2786(A→C)	929(E→A)		
	2803(A→G)	935(T→A)		
GFL101	2046(A→C)	682(Q→H)	0.19	++±
	2630(G→C)	877(R→P)		
	2678(T→C)	893(V→A)		
	2786(A→C)	929(E→A)		
	2849(A→C)	950(Q→P)		
Wild type			0.016	++

-The data presented are representative of three independent experiments.
